# Intrinsic chiral photoswitches manipulate soft-materials

**DOI:** 10.1038/s41377-022-00785-w

**Published:** 2022-04-14

**Authors:** Jihong Yu

**Affiliations:** State Key Laboratory of Inorganic Synthesis and Preparative Chemistry, College of Chemistry, Jilin University, Changchun, P. R. China

**Keywords:** Optics and photonics, Optical materials and structures

Nature Photonics **16**, 226–234 (2022) 10.1038/s41566-022-00957-5

Advanced Materials **34**, 2110170 (2022) 10.1002/adma.202110170

Due to the lack of appropriate reversible photoswitches, efficiently remote photoprogramming of self-organized soft helical superstructures represents a formidable challenge in particularly allowing multiple stable states, tuning over a broad spectral range and establishing coupling balance between photonic resonance and transmission. The team of Weihong Zhu, Zhigang Zheng and Ben Feringa brings forth a series of exciting light-switchable intrinsic chiral diarylethenes with extremely large helical twisting power (HTP) to enable the dynamic, helical, and optical microstructure manipulation of liquid crystals (LCs), unprecedentedly achieving controllable, selectable, and extractable multi-stable reflection states, meanwhile avoiding the inherent orientation disorderliness of LCs caused by multiple chiral sources. A cutting-edge multiple anti-counterfeiting technique, featuring color-tunability, erasability, reversibility, multi-stability and viewing-angle dependency of pre-recorded patterns, has been well demonstrated with the overwhelming intrinsic chiral superstructures. The new exciting strategy can create a gorgeous landscape of optical information processing and configuring, and even build up a unique laser in quadri-dimensional manipulation of wavelength, wavefront, spin angular momentum, and orbital angular momentum, endowing a sharp and narrow band-width with both remarkable thermodynamic stability and robust fatigue-resistance.

